# The Myth of the Placebo Response

**DOI:** 10.3389/fpsyt.2019.00577

**Published:** 2019-08-16

**Authors:** Wayne B. Jonas

**Affiliations:** ^1^Samueli Integrative Health Programs, Alexandria, VA, United States; ^2^Georgetown University School of Medicine, Washington, DC, United States; ^3^Department of Family Medicine, Uniformed Services University, Bethesda, MD, United States

**Keywords:** placebo, myth, response, healing, traditional

## Abstract

The placebo response is a myth. It does not exist in reality, and continuing to name it is hindering the optimal application of science to healing in medicine. On the surface, it is obvious that, when defined as a biological response to an inert pill (like a sugar pill), the idea of a “response” to a placebo is impossible. Inert treatments by definition do not produce responses. So why do we continue to ponder why people get better from taking inert substances and base our acceptance of legitimate treatments on demonstrating that they go beyond that response? The problem arises because we have flawed assumptions of the value that reductionistic science and the demonstration of specific effects has for healing. To support those flawed assumptions, we support the idea of “the placebo response.” This causes confusion among patients, clinicians, regulators, and even scientists. Legitimate medical treatments have become defined as those that do more than produce a placebo response. An entire pharmaceutical industry and its regulators attempt to control and profit by proving that small molecules produce a clinical effect greater than the placebo response. Billions of dollars are made when that is proven, often even when the size of the response in the active over the placebo group is miniscule. The fact is people heal and that inherent healing capacity is both powerful and influenced by mental, social, and contextual factors that are embedded in every medical encounter since the idea of treatment began. In this chapter, I argue that our understanding of healing and ability to enhance it will be accelerated if we stop using the term “placebo response” and call it what it is—the meaning response, and its special application in medicine called the healing response.

## Traditional Healing Systems

For millennia, the primary philosophy behind most healing traditions involved seeking balance and harmony with your spiritual self, social community, and nature (1). Patients and practitioners in these traditional systems adjusted how the patient lived in the society, with nature and with themselves, the latter referring to the spiritual and mental aspects of life. Hippocrates said that the physician’s highest task was supporting the patient while nature did the healing—*Vis medicatrix naturae*, literally “the healing power of nature (2).” The Yellow Emperor of China talked about the physician working to keep the patient healthy through balance with nature and lifestyle (3). The ancient Ayurvedic system of medicine involves returning the patient to the unity of wholeness of a human being—called universal consciousness—as the path to induce healing processes (4). In most of these ancient healing traditions, the mind, heart, body, and nature are considered all one, and health came from getting them to work in harmonious interaction. Traditional healing systems from around the world kept their eye on the whole picture of the human person, which was defined as an individual in the context of their social and natural environment.

## Western Biomedicine

Then, approximately 150 years ago, some things were discovered in the Western hemisphere about the small and particular that radically changed this thinking. The microscope and chemistry were invented, and we began to identify and isolate bugs (infectious agents) and drugs (chemicals) as causes and cures for certain diseases. Manipulating these smaller elements had a dramatic ability to stop death from those causes. These discoveries worked particularly well for infectious disease and trauma, which were the primary causes of immediate death for the millennia before that. So dramatic where these effects that a new Western version of medicine grew up, which rapidly spread and globally supplanted the older healing traditions. After all, who would not want to have their life saved when they were on the verge of death? And so, like cars and cell phones after them, Western medicine became the dominant system throughout the world backed by policy, payment, and delivery. The age of heroic medicine had arrived. Nature was now to be dominated and controlled. The idea of harmony and balance went out the window. The more holistic models from ancient times were swept away or were relegated to the so-called complementary or alternative medicine (CAM) practices. These ancient traditions were called “non-scientific” and delegitimized. Since Western medicine was particularly focused on the physical, no longer was the mind, spirit, or social dimensions of the person relevant for healing. No longer was the healing force of nature important. These concepts, previously foundations across the globe, largely disappeared from the medical encounter (5).

## The Rise of Chronic Disease

Except that disease did not disappear. It only shifted. Our ability to stop death resulted in an aging population and the emergence of chronic diseases as the dominant causes of morbidity and mortality. Diseases such as heart disease, diabetes, depression, dementia, obesity, and cancer now dominate humanity (6). These diseases do not respond well to the Western science of the small and particular. However, by now, we believed so much in this science and have seen its dramatic effects in acute disease and death that we continue to use this model and apply it to chronic illness. Our research is now organized around identifying the specific physical causes of chronic conditions, which has become the main criteria for what is legitimate or illegitimate practice. The science of the small and particular is imbedded in regulatory processes for approving and paying for treatments. The medical industry follows these regulations and seeks approval of proprietary small molecules for common chronic diseases. Billions of dollars flow following these approvals. Drugs—as defined by regulatory and patent bodies—dominate medical thinking and practice.

## Reductionism

However, most of these approaches do not work very well. The evidence is now abundantly clear that, at least for the management of complex chronic diseases, reductionism does not work well for and is inferior to whole systems approaches in practice. This can be illustrated in a number of ways. First, the narrow, reductionistic view is the underlying reason the pharmaceutical industry invests up to two billion dollars and takes 12–15 years to get a new drug on the market^1^,^2^. The vast majority of drugs fail when ultimately tested in large studies compared to placebo treatments. Many those that are proven and do get on the market don’t work very well. Two thirds of the positive research published in the mainstream literature cannot be replicated (7–10). For those that can be replicated, the effect size—that is, the effect of the drug group over the placebo group—is small. In a recent study, researchers at the United States National Health, Lung and Blood Institute analyzed the benefit of the medications that it funded research on for heart disease over the last 30 years. The result was that these drugs added ∼8% over the spontaneous or placebo healing rates for those diseases (11).

Even simple proven and effective therapies such as statins for the prevention of heart disease illustrate this dilemma further. For every 100 people who take a statin for the primary prevention of heart disease, only two will avoid a heart attack by doing this, 98 will derive no benefit (but we or they have to pay for the drug), and 5–20 will suffer significant side effects. To get these small benefits, many must tolerate these side effects and costs. Who determines whether this benefit is better than the harms? That is not a scientific question, it is a value question that each patient and their physician must make for themselves (12). Unfortunately, physicians are armed almost completely with the tools that industry provides them. Rarely is a decision about statin use offered in the full context of the benefits and costs of alternative approaches such as behavior, the ritual of compliance, social and emotional factors such as loneliness, or the impact of patient and cultural beliefs and expectancies.

The recent promise of “personalized, precision medicine”—the ultimate extension of the reductionistic approach—in an attempt to control even more specific molecular targets—is also, so far, largely a disappointment, although hope and hype spring eternal in this field. Precision (targeted) oncology is the most developed of these approaches. There have been some dramatic effects in certain people from hitting these targets with small molecules. Precision oncology has produced dramatic benefits (and major harms) in small populations. However, the promise of these breakthroughs for large populations is, overall, modest and overhyped. Professor Dimitrios Roukos, from the Personalized Cancer Medicine Biobank, Ioannina University School of Medicine in Greece summarized this as follows: “… *the results of clinical trials testing biomarkers and biologics developed on the basis of conventional single-gene cancer research have demonstrated modest, isolated clinical success. These findings are not surprising given the molecular network complexity and heterogeneity of cancer. In the post-genomic era, next-generation DNA-sequencing technology-based results confirm available evidence that cancer initiation, growth and metastasis are driven by molecular networks rather than just one mutated gene or a single deregulated signaling pathway* (13).” What is needed is not only simply a personalized, precision oncology, where the drug is targeted to a unique molecule on a cell, but also a reversed personalized oncology, where the patient is adjusted to enhance the drug response. This requires a more holistic view than the current paradigm of the small and particular provides.

## Invention of the Placebo Response

Since reductionism has largely failed for chronic illness, yet Western medicine is already heavily invested in it, both in mindset and money, health care had to invent a way of solidifying its legitimacy further. Thus, it invented the “placebo response.” Into the term placebo response was dumped all the rest of healing that was not produced by the isolated, physical, and specific treatment. Being seen as not placebo—meaning being specific and physical—became the requirement to be considered valid and real (14). Effects that could not demonstrate they were due to a specific and physical entities were said to be “just placebo” and therefore not real and not valuable for healing. Relegating effects to placebo provided a way to cover up the fundamental flaw in the reductionistic model—that it does not work for healing complex, multi-factorial, chronic disease.

## Clearing the Placebo Myth

While the solution to this dilemma is multifaceted, one important step would be to stop pursuing the mythical concept called the placebo response. Several years ago, Professor Dan Moerman and I recommended that we replace the term placebo response with the term the “meaning response (15).” The reason for this was to make it more evident that our physiology was responding to the context and rituals that imbued meaning to a treatment rather than to a substance, inert or otherwise. And while the meaning response framework has gained some traction, it too was unsatisfactory for motivating the transformation needed in the medical encounter. While I still believe the term “meaning response” should replace the concept of “placebo response,” we should also replace the words “placebo response” with the words “healing response” when referring to the use of meaning in treatment. This would acknowledge that it is the whole person that is in need of medicine taking into account the underlying mechanisms that produce those responses rather than attributing them to placebo. By abandoning the concept of a placebo response, we could bring into focus how our mind and expectations alter our biology and how the cultural rituals and environmental context of medicine induce maximum healing through meaning, rather than defaulting into debates over whether a treatment effect is “real” or “just placebo” based only whether it works through a specific theory or a small molecule.

Making this conceptual and linguistic shift would change the entire nature of placebo research for health care. Suddenly, research on the meaning or healing response and its mechanisms would become more valuable for use in practice. Rather than simply using placebo-controlled research to eliminate what is “not real”—a consequence of the placebo myth that has left us with a paucity of proven therapies for chronic disease—research on how the meaning response works opens us up to an abundance of discoveries that can be immediately applied in practice. What is now dismissed as the placebo response could be used as the basis for inducing optimal healing that is personalized to the patient and their culture and context. We would rapidly go from therapeutic nillism to an abundance of ways to alleviate suffering and treat chronic disease.

## Releasing Practice from the Placebo Myth

By clearing away the placebo myth, I, as a physician, can use the understanding of the mechanisms of the meaning response to construct multiple paths for healing my patients. I can widen my therapeutic lens. For example, I can now use the power of mindset and belief to heal. I can create social rituals for healing that are specific for a patient and their culture. I can adjust the environment of the patient to optimize healing. I can value and use the doctor–patient relationship again—which has largely lost its place in Western medicine, and I can also use this knowledge to avoid harm, the so-called nocebo response. Destroying the placebo myth returns meaning to medicine, brings hope to the patient, and allows me to address the root causes of recovery. In addition, it could potentially reduce burnout by returning the heart of medicine—relationships—back into healthcare. Research on the meaning response and how it can be applied to healing would take us from looking at the effects found when using inert substances as simply curiosities to a new fundamental way for understanding how to optimize therapeutic practice.

Once the myth of the placebo response is removed, I, as a physician, can draw on research on the mechanisms of the meaning response to produce an evidence-based healing response for my patients. For example, I would now have evidence for using the following approaches in my day-to-day practice with any treatment, no matter what its efficacy is. I would try to use more frequent dosing rather than less frequent dosing—up to a limit (16). I would seek to deliver therapies in the most powerful therapeutic settings such as hospitals and clinics rather than at home (17). I would try and match the appearance, such as size and color, to the desired effect expected by the patient and their culture (16, 18). I would attend to the style and route of administration of a treatment (17). I would take the time to deliver therapies in a warm and caring way (19) and with confidence in their power to heal (20). I would explore what therapies my patient believes in and try to align and accommodate my treatment to that belief, provided it was safe (21–23). I would make sure I understand the mechanisms of a treatment so that I can believe in the treatment I am delivering (24, 27). I would seek to align all beliefs—that of the patient, the doctor, the family, and the culture (25). I would add a safe and easy to use conditioned stimulus alongside the specific therapy (26, 27). I would use a well-known brand or a new and exciting treatment claimed to have success (28–30). I would let the patient know what to expect (31, 32). I would seek to use an electronic device to deliver and track the treatment when possible (28). I would always incorporate reassurance, relaxation, suggestion, and reassurance into the treatment (33–35). I would spend the time to listen and understand the patient (19, 36) and, when possible, touch them with empathy and reassurance (15, 37). More recently, the evidence shows that I can simply explain to the patient about the likely benefit of any treatment for its potential in healing and recovery (38, 39), and most remarkably, I can do this with any treatment, whether its specific effect has been proven or not.

## Releasing Research from the Placebo Myth

Getting rid of the placebo myth also brings a breath of fresh air to biomedical research in general. First, we can alter our research designs to reduce the meaning response in the early phases of clinical testing and thus widen the gap between the effects of meaning and the medicine (40). This would allow us to demonstrate the specific effect of a treatment more easily, with fewer subjects and less expensively. In addition, it would help us build a basis for advancing both the evidence and ethical foundations for using meaning in medicine (41).

Freed from the placebo myth, we are no longer bound to an outdated hierarchy of evidence for determining what is valid and valuable. We can now structure our research agenda around what is useful for the patient (42). I call this patient-centered science. Safety comes first. If a treatment is safe with unknown efficacy, we still have the ability to use it in the care of the person for their benefit by optimizing the meaning effect. Recovery becomes more prominent. Rather than finding a molecule that I must give life-long to hold down a specific physiological mechanism deemed to be pathological, I can look for treatments that are stimulatory—inducing a more durable and low risk healing response. For example, rather than adding three drugs onto an antihypertensive regimen (the current stepped care standard), I can approach the patient with diet or exercise or meditation or acupuncture to treat their blood pressure and heal it at its root causes^3^. With this abundance of healing response tools now established as safe and effective, my ability to personalize a treatment regimen becomes more flexible and doable for a patient. If a drug produces side effects or cost too much for an individual patient, I can approach them through lifestyle and diet or through mind–body practices or conditioning or through a variety of a traditional and complementary approaches previously shown to be safe (43).

Finally, freed from the myth of the placebo response, our medicine and our science align with the reality of the complex ecological system that is a whole person (44). We now can fit the ecological complexity with complexity science. This has been known for decades by the term the biopsychosocial model (45). In complexity science, the parts do not explain the whole, and they are not additive. Instead, once the complexity of the parts gets to a certain point, there emerge new properties with new dynamics. Complexity science—the science of the large and the whole—provides an evidence base for treating a patient through multiple methods at the level of mind, body, social, or spirit (46). The translational gap between science and practice is now shortened. No two billion dollars and 15 years required for validity. Finding meaning opens multiple approaches to healing supported by an array of research methods.

## Making the Healing Response Routine

**Figure 1:**
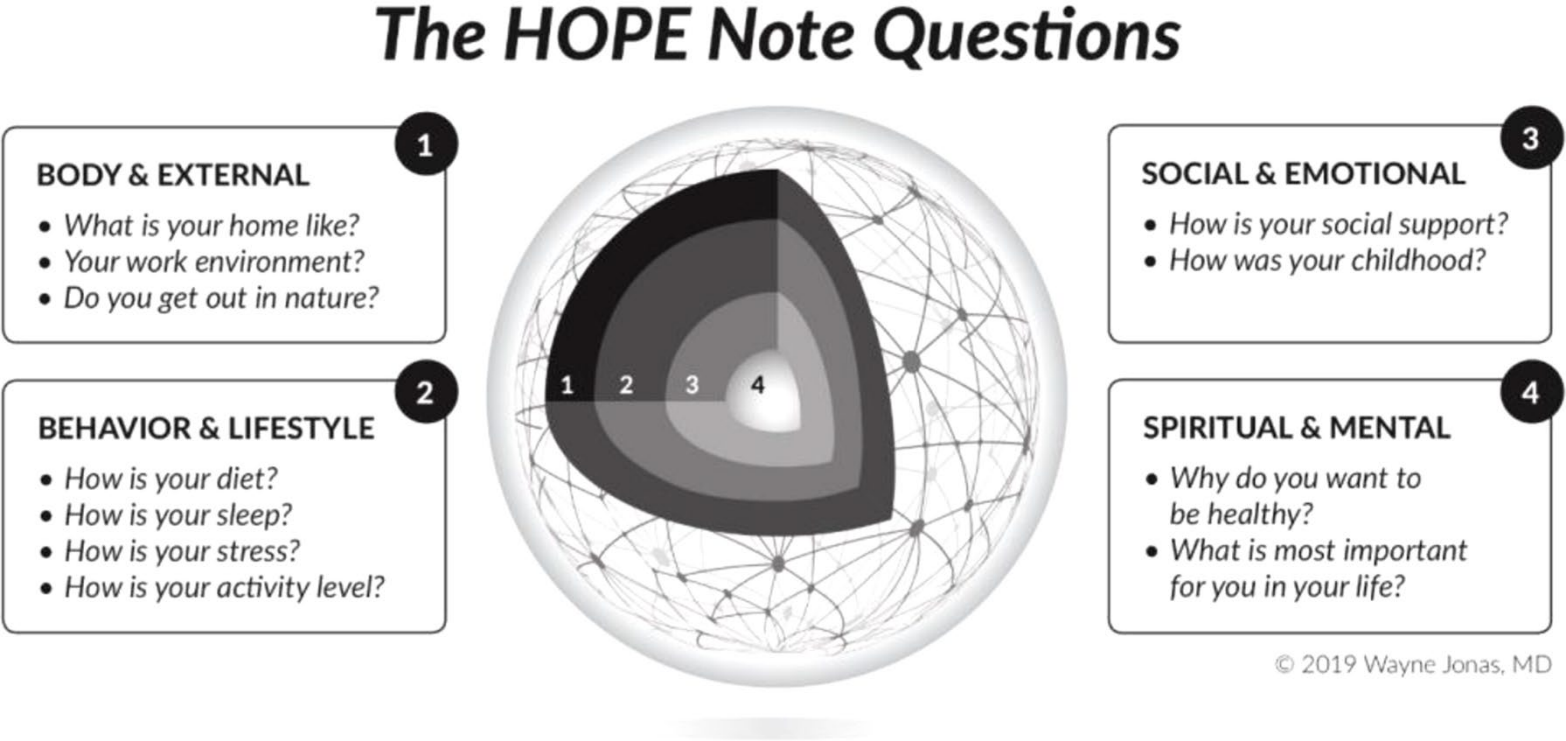
The HOPE Note starts with the central question exploring “What Matters” in a patient’s life that would help them attain and maintain health and wellbeing (#4). It then explores the patient’s personal determinants of health including: their social and emotional life (#3), their lifestyle and behavior (#2), and the physical environment in which they work, play, and live (#1).

Once freed from the placebo response myth, how can we use this newfound evidence from complexity science to heal? **Figure 1** llustrates a four-dimensional model of a person that I use in my practice to routinely enhance healing, based on knowledge from the meaning response that is derived from research using placebo treatments.

I do this through a set of questions and assessments that I call the HOPE Note (47). HOPE stands for healing-oriented practices and environments and draws heavily from looking at the placebo arms of controlled clinical trials and laboratory studies that illuminate the mechanisms of the meaning response. In the HOPE Note, I begin by asking the patient what matters to them in their life—why are they living and why do they want to have health. This makes finding meaning the central goal of the encounter and the interchange person centered from the beginning. We then go on to explore the multiple ways in which a healing response can be induced through mind–body practices, or through the social and emotional environment, or through lifestyle, or by altering the physical context in which treatment occurs. Knowledge from research using placebos and unpacking the meaning response infuses those discussions with a solid evidence base and helps the patient optimize and personalize their healing^4^.

Eliminating the myth of placebo will not be easy. Currently, medical care derived from the science of the small and particular provides us with only about 15–20% of the health benefits for populations, yet it gets 80–90% of the money (48). Our inherent healing response as accessed through behavior and the social environment accounts for the other 80%. However, this approach to illness has no business model to drive it forward or make it accessible to everyone. Even more difficult than changing the economic model of healing will be changing our minds about how healing works. A good first step would be to see the placebo response for what it is—a conceptual myth that sustains a broken medical system and covers up what we are really seeking—our inherit healing capacity now freed by understanding how deeply meaning infuses us all.

## Author Contributions

The author confirms being the sole contributor of this work and has approved it for publication.

## Conflict of Interest Statement

The author declares that the research was conducted in the absence of any commercial or financial relationships that could be construed as a potential conflict of interest.

## Footnotes

1 https://www.drugs.com/fda-approval-process.html

2 https://www.washingtonpost.com/news/wonk/wp/2014/11/18/does-it-really-cost-2-6-billion-to-develop-a-new-drug/?noredirect=on&utm_term=.87ac8cbe951c

3 https://www.mayoclinic.org/diseases-conditions/high-blood-pressure/in-depth/high-blood-pressure/art-20046974

4
http://drwaynejonas.com/resources/hope-note/
